# Analytical Scheme for Simultaneous Determination of Phthalates and Bisphenol A in Honey Samples Based on Dispersive Liquid–Liquid Microextraction Followed by GC-IT/MS. Effect of the Thermal Stress on PAE/BP-A Levels

**DOI:** 10.3390/mps3010023

**Published:** 2020-03-24

**Authors:** Ivan Notardonato, Sergio Passarella, Giuseppe Ianiri, Cristina Di Fiore, Mario Vincenzo Russo, Pasquale Avino

**Affiliations:** 1 Department of Agriculture, Environmental and Food Sciences, University of Molise, via De Sanctis, I-86100 Campobasso, Italy; ivan.notardonato@unimol.it (I.N.); sergio.passarella@studenti.unimol.it (S.P.); g.ianiri@studenti.unimol.it (G.I.); c.difiore@studenti.unimol.it (C.D.F.); mvrusso@unimol.it (M.V.R.); 2 Institute of Ecotoxicology & Environmental Sciences, In-700156 Kolkata, India

**Keywords:** analytical protocol, phthalates, bisphenol A, DLLME, GC-IT/MS, ultrasound, dispersive solvent, honey, honeycomb, thermal stress

## Abstract

In this paper, an analytical protocol was developed for the simultaneous determination of phthalates (di-methyl phthalate DMP, di-ethyl phthalate DEP, di-isobutyl phthalate DiBP, di-*n*-butyl phthalate DBP, bis-(2-ethylhexyl) phthalate DEHP, di-*n*-octyl phthalate DNOP) and bisphenol A (BPA). The extraction technique used was the ultrasound vortex assisted dispersive liquid–liquid microextraction (UVA-DLLME). The method involves analyte extraction using 75 µL of benzene and subsequent analysis by gas chromatography combined with ion trap mass spectrometry (GC-IT/MS). The method is sensitive, reliable, and reproducible with a limit of detection (LOD) below 13 ng g^−1^ and limit of quantification (LOQ) below 22 ng g^−1^ and the intra- and inter-day errors below 7.2 and 9.3, respectively. The method developed and validated was applied to six honey samples (i.e., four single-use commercial ones and two home-made ones. Some phthalates were found in the samples at concentrations below the specific migration limits (SMLs). Furthermore, the commercial samples were subjected to two different thermal stresses (24 h and 48 h at 40 °C) for evidence of the release of plastic from the containers. An increase in the phthalate concentrations was observed, especially during the first phase of the shock, but the levels were still within the limits of the regulations.

## 1. Introduction

In recent years, the goal of researchers in the field of analytical chemistry has been both to develop really sensitive analytical protocols, but also, and above all, simplify analyses considering very important factors such as time and cost. For instance, the analysis time is currently considered the limiting process of the analyses. Basically, most people are only interested in purely economic costs without thinking that they are also connected to environmental ones. The main idea is therefore to use miniaturized analytical techniques for reducing the costs whereas, with regard to the time factor, efforts are being made to develop techniques that accelerate the sample treatment. Over the years, it has passed from liquid–liquid extraction (LLE) [1] to a solid-phase extraction (SPE) [2,3] up to developing a new technique, dispersive liquid–liquid microextraction (DLLME), which uses very few quantities of solvents when compared to those of the LLE and which eliminates the problems connected with the SPE [4]. The DLLME, developed in 2006 by Razaee [5], made a notable innovation in the field of extraction analysis. Due to its simplicity, rapid operation, low solvent consumption, and a modest request for instrumentation, it has spread widely in the analytical field [6,7].

DLLME is based on a ternary system in which the extraction solvent and the dispersion solvent are quickly added to the sample in aqueous form, creating a cloudy solution. In this way, the micro extractant droplets are conveyed by the dispersing solvent throughout the solution, thus creating a large contact surface between the extraction solvent and the sample. The extraction solvent is separated by centrifugation and withdrawn with a micro syringe for chromatographic analysis, mainly Gas Chromatography (GC) [8]. In the beginning, after centrifugation, high density chlorinated solvents were used to obtain better sedimentation and separation of the extraction droplets [9]. However, growing concerns about the toxicity and limitations of typical chlorinated solvents have favored the use of less toxic but lower density compounds such as long chain alcohols or hydrocarbons [10]. Since the droplets float on the surface of the aqueous sample after extraction, various studies have focused on their collection [11]. The dispersion methods used, aside from the use of the dispersive agent, can be different in the analytical DLLME technique. For instance, one dispersion technique is temperature-controlled dispersive liquid-phase microextraction (TC-DLPME) [12], which can only be applied when the solubility of the extraction solvent is a function of temperature: a change in temperature greatly changes the solubility of the solvent, which mixes completely with the aqueous solution of the sample [13,14]. Another dispersion technique is ultrasound dispersive liquid–liquid micro extraction (US-DLLME), which uses ultrasound energy to develop the emulsion process of the extraction solvent in the sample solution without any dispersive solvent [15,16]. To facilitate the dispersion of the extraction solvent, it is also possible to use the surfactant assistant dispersive liquid–liquid micro extraction (SA-DLLME) where the dispersive solvent is replaced by a surfactant which, by reducing the surface tension of the extraction solvent, allows for a greater and more stable contact surface with the analytes present in solution [17]. DLLME has been widely applied for analyzing environmental samples [18,19,20], but has not been considered highly compatible for the extraction of analytes from complex matrices such as food. In fact, foods show a marked matrix effect and due to the potential interaction of the components of the matrix in these samples with organic solvents, the extraction phase becomes complicated when obtaining a suitable substrate for analysis [11]. For semi-solid foods, it is possible to opt for an initial freeze-drying of the sample, which is then re-dissolved in water, avoiding problems of phase separation following centrifugation [21].

Phthalates (PAEs) and bisphenol A (BPA) are important chemical building blocks in the plastics industry. They are also ubiquitous contaminants in the human body, wildlife, and the environment [22,23,24]. PAEs are chemicals that are often used as softeners for polyvinyl chloride (PVC) plastic. To make plastic more flexible, plasticizers are needed, and they are in most cases phthalates. However, some PAEs are harmful to your health: some of them are classified as endocrine disruptors that are toxic to reproduction, which means that they may damage fertility or the unborn child. BPA is a substance that is used in the industrial manufacture of polycarbonate plastic products. These include common consumer goods such as re-usable plastic tableware and bottles for drinks, sports equipment, compact discs (CDs), and digital versatile disc (DVDs). Epoxy resins containing BPA are used to coat the inside of water pipes and the inside of cans for food and drink to increase their shelf-life and avoid obtaining a metallic taste on the food or drink. It is also used in the coating of sales receipts. BPA has also been classified as an endocrine disruptor. As such, there are many plastics that are unsafe for food storage and processing. For instance, different labs have tested honey samples for the presence of microplastics along with pesticides and other detrimental chemical residues.

The aim of this paper was to develop a fast, sensitive, and reproducible method for the analytical determination of PAEs and BP-A in real matrices, with particular attention to honey. Through both pollen and nectar foraged by flowers and water, bees daily collect the residues of contaminants present in the environment. Often, precisely for these reasons, bee products and to a lesser extent honey, are used as monitors (indicators) of environmental contamination. Furthermore, in recent years, the use of plastic honeycombs has spread to reduce the risk of melting the wax during the hottest summer seasons, with consequent loss of the crop. The possible presence of PAEs and BPA in honey is also related to the production processes that may involve direct contact with unsuitable plastic (e.g., single-dose plastic packs).

The determination of PAEs and BPA presents considerable difficulties related mainly to their low concentration, which is currently difficult to determine even with very sensitive instruments [25]. To overcome this problem, it is necessary to develop an analytical method that provides for the extraction and pre-concentration procedure. In this paper, ultrasound vortex assisted DLLME (UVA-DLLME) was used as the extraction technique [26], whereas Gas Chromatography coupled with Ion Trap Mass Spectrometry (GC-IT/MS) was used as the analysis technique. The method developed, validated, and experimentally consolidated in the laboratory takes into account all the parameters that influence the extraction of the analytes. For this purpose, the extraction solvent, the ionic strength of the solution, the pH, and the extraction volume were analyzed and studied.

The developed method was applied to six real samples (i.e., four single-use commercial ones available in the Italian market, one home-made honey nectar sample, and one sampled from honey produced in plastic honeycomb). This last sample is interesting because the plastic honeycomb represents a new frontier in honey production, according to the author’s knowledge, this measurement is the first analysis for understanding the migration of such compounds from the honeycomb to the honey. Finally, the four commercial samples were subjected to thermal stress (i.e., exposed to 40 °C for 24 and 48 h) for evidencing the effects of the heat on the plastic containers.

## 2. Experimental Design

The investigated compounds are listed in Table 1, along with the main analytical parameters.

### 2.1. Chemicals

- Dimethyl phthalate (Sigma-Aldrich, Milan, Italy; Cat. no.: 41320; purity ≥99.5%)
- Diethyl phthalate (Sigma-Aldrich, Milan, Italy; Cat. no.: 53008; purity ≥99.5%)
- Diisobutyl phthalate (Sigma-Aldrich, Milan, Italy; Cat. no.: 43540; purity ≥99.9%)
- Dibutyl phthalate (Sigma-Aldrich, Milan, Italy; Cat. no.: 36736; purity ≥98%)
- Bis(2-ethylhexyl) phthalate (Sigma-Aldrich, Milan, Italy; Cat. no.: 36735; purity ≥99.9%)
- Di-*n*-octyl-phthalate (Sigma-Aldrich, Milan, Italy; Cat. no.: 80153; purity ≥98.0%)
- Bisphenol-A (Sigma-Aldrich, Milan, Italy; Cat. no.: 42088; grade certified reference material)
- Phenanthrene (C_14_H_10_; Lab Service Analytica, Anzola Emilia, Bologna, Italy; Cat. no.: CILDLM3711)
- *n*-Heptane (Carlo Erba, Milan, Italy; Cat. no.: 446841)
- *iso*-Octane (Carlo Erba, Milan, Italy; Cat. no.: 456734)
- Toluene (Carlo Erba, Milan, Italy; Cat. no.: P0710540)
- Benzene (Carlo Erba, Milan, Italy; Cat. no.: 426113)
- Acetone (Carlo Erba, Milan, Italy; Cat. no.: 508200)
- Sodium chloride (Carlo Erba, Milan, Italy; Cat. no.: 368257000)

### 2.2. Standard Solutions

- Preparation of stock solution for each analyte, 1000 µg g^−1^:
  ○ weigh 10 mg of each PAE/BPA;
  ○ make up to volume with 10 mL of acetone.
- Preparation of diluted PAE/BPA mix solution, 10 µg g^−1^:
  ○ appropriate dilution of the mother solutions with acetone to set up a PAE/BPA mix solution.
- Preparation of the solutions for the calibration curves:
  ○ appropriate dilution of the PAE/BPA mix solution, 10 µg g^−1^, with acetone to obtain seven solutions of known concentrations (0.03, 0.05, 0.1, 0.25, 0.5, 1.0, and 5.0 µg g^−1^).
- Preparation of the phenanthrene (Internal Standard, I.S.) stock solution:
  ○ weigh 1 mg of phenanthrene;
  ○ make up to volume, 10 mL, with acetone (100 µg g^−1^);
  ○ appropriate dilution for obtaining 0.5 and 0.05 µg g^−1^ I.S. solutions.
- All solutions were stored in darkness vials at 4 °C.

### 2.3. Equipment

- Gas chromatograph (GC) TraceGC (ThermoFischer, Milan, Italy; Cat. no.: MS210649)
- Ion Trap Mass Spectrometry (IT/MS) PolarisQ (ThermoFischer, Milan, Italy; Cat. no.: MS210649)
- Software Xcalibur, version 1.4.1 (ThermoFischer, Milan, Italy; Cat. no.: 1.4.1 SR1)
- Fused-silica capillary column, 95% dimethylpolysiloxane - 5% phenyl, 30 m × 0.25 mm × 0.25 μm (Teknokroma, Rome, Italy; Cat. no.: TRB-5MS)
- Centrifuge Neya 8 (Giorgio Bormac S.r.L., Carpi, Italy; Cat. no.: ZBDN-04729)
- Ultrasounds Starsonic 18-35 (Liarre s.r.l., Casalfiumanese, Italy)
- Vortex ZX3 (VELP Scientific, Usmate, Milan, Italy; Cat. no.: F202A0176)

## 3. Procedure

### 3.1. Protocol for Phthalates and Bisphenol A (PAEs/BPA) Analysis in Honey Samples

1. Weigh 2.5 g of honey.
2. Add distilled water up to 10 g.
3. Solubilize the honey in the solution.
4. Check pH and adjust at pH 4. 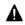 **CRITICAL STEP**: the pH choice is decisive for the successful procedure. PAEs are better extracted at pH alkaline but they are only recovered at pH 4, BPA is extracted at acid pH whereas at alkaline pH its recovery is very low (between 20–30%): pH 4 allows for the quantitative recovery of all compounds.
5. Add 7.5 µL of phenanthrene as I.S.
6. Vortex 15 s.
7. Add the extraction solvent, 75 µL of benzene.
8. Vortex 5 min: formation of the macroemulsion.
9. Ultrasound 6 min. 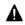 **CRITICAL STEP**: This step is fundamental for the formation of the microemulsion. The ultrasound give the power for the microemulsion.
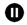
**PAUSE STEP**: the microemulsion formation is essential for the procedure. If it does not form, the analytical procedure can be stopped because the extraction has not occurred.
11. Add NaCl 10 g L^−1^ to break the microemulsion. **OPTIONAL STEP**: the addition of NaCl helps the microemulsion break.
12. Centrifugation at 4000 rpm per 30 min.
13. Withdraw 1 µL of the supernatant.
14. Inject into gas chromatography equipped with ion trap mass spectrometry (GC-IT/MS).

### 3.2. Thermal Stress Procedure

- 24 h at 40 °C, withdrawing 2.5 g of honey and processing like in Section 3.1.
- 48 h at 40 °C, withdrawing 2.5 g of honey and processing like in Section 3.1.

## 4. Results and Discussion

The first applications found excellent results in the pre-concentration of organic analytes from aqueous samples, demonstrating high extraction efficiency [27,28,29]. However, before proceeding with the extraction of the investigated analytes, some fundamental parameters must be optimized for the correct extraction. Among these, the most important are the volume of extraction and dispersive solvent, the extraction time, the pH, the ionic strength, and the amount of sample examined. All optimization procedures were carried out on a blank honey sample spiked with known amounts of PAEs/BPA. A blank sample of honey was collected from a local apiary and stored in darkness 4 °C in PAE-free glass bottles: it was processed in advance for the optimization of extraction conditions and validation of the developed method.

The pH of the aqueous solution is decisive for the quantification of the molecules because at basic pH, following the centrifugation phase, there is the formation of a gel, which by absorbing the analytes adversely affects the recoveries. Furthermore, the natural composition of most honeys allows for an easier pH adjustment at acid values without drastically changing the nature of the matrix [30].

The ultrasound treatment of the solution is a critical step because this allows for the formation of the oil-in-water microemulsion. This occurrence makes the solvent droplets very small, which disperse in the solution, thus increasing the contact surface and recovery.

The addition of NaCl favors the reverse of the phases and therefore the breaking of the emulsion; it also increases the ionic strength of the solution, further promoting the extraction of the molecules by reducing the solubility of the analytes (salting-out effect) [31]. With regard to the extraction of the molecules, studies in this laboratory showed that even without the addition of NaCl, the recoveries were quantitative [32,33,34]. Considering the matrix in question, the solution also had good ionic strength due to the presence of mineral salts, even in the absence of NaCl.

To identify the best extractant solvent, five different solvents were tested (i.e., *n*-heptane, *iso*-octane, benzene, toluene, and toluene + acetone (1 + 1 v/v). The recoveries are reported in Table 2, benzene showed the best performance in the compound extraction.

The choice of benzene as the extraction solvent deserves a reason because it is well-known for its dangerousness (i.e., potential occupational carcinogen, flammability. The International Agency for Research on Cancer, IARC, rated benzene as “known to be carcinogenic to humans”, Group 1). Although it is more hazardous than the other solvents tested, it demonstrated almost quantitative recoveries for all the molecules under study, especially BPA, and not only for some of them such as has occurred for toluene, iso-octane, and heptane. In addition, the use of benzene has allowed competitive or even better Relative Standard Deviations (RSDs) to be obtained than the other papers present in the literature. The risks for the operator can be reduced by adopting both suitable personal protective equipment (PPE) and collective protection equipment (CPE), and by carrying out the critical operations under a fume hood. Furthermore, in the proposed method, only a very small amount of benzene (75 μL) is needed. This last issue (i.e., working with small volumes) also involves easy disposal management for environmental protection.

Afterward, the volume of the extractant solvent and the best pH of the solution were investigated. Six different volumes (25, 50, 75, 100, 150, and 200 µL) were considered and the relative recoveries were compared: Table 3 shows that the best recoveries were obtained using 75 µL of benzene whereas Table 4 shows that pH 4 allowed for excellent recoveries to be obtained for all of the analyzed compounds.

Optimal times and rotation times for vortexing (5 min), ultrasound (6 min), and centrifugation (30 min at 4000 rpm) were established on the basis of previous studies carried out in this laboratory [21,32].

**Figure 1:**
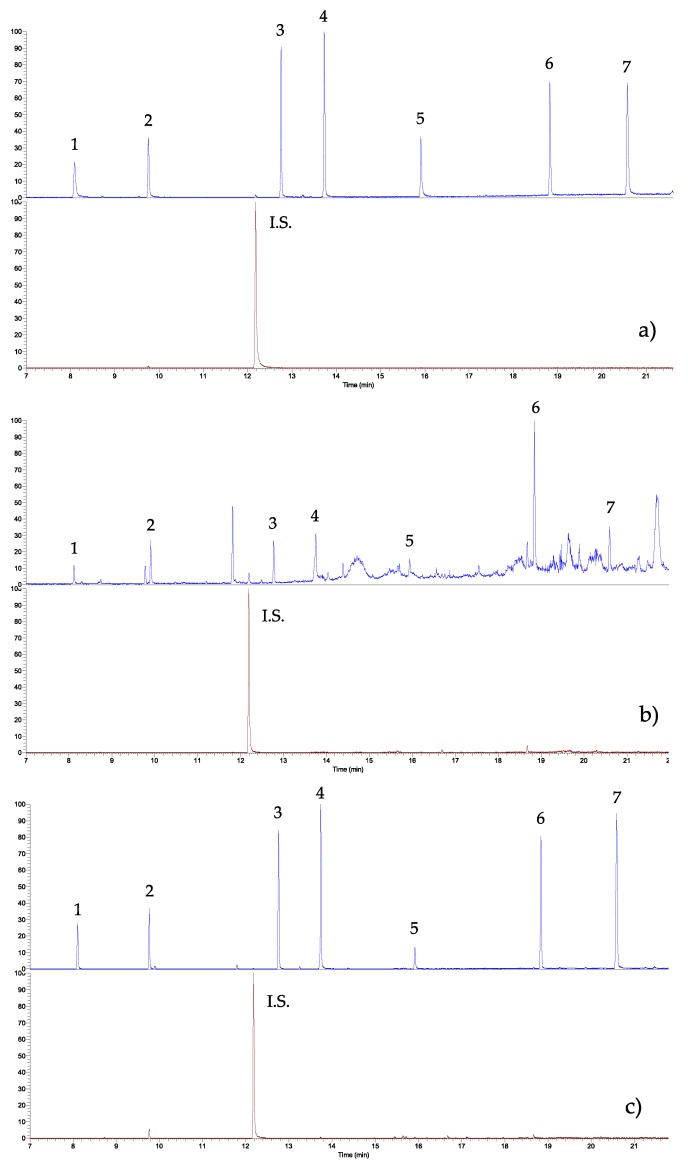
GC-IT/MS chromatograms of both (**a**) the standard PAEs/BPA mixture solution, (**b**) honey sample, and (**c**) the same honey sample spiked with the standard solution (each PAE and BPA at 200 ng g^−1^; 75 μL of benzene as extraction solvent, pH 4, vortex mixing for 5 min, ultrasonication for 6 min, 25 °C, centrifugation for 30 min at 4000 rpm; and NaCl at 10 g L^−1^). For the experimental conditions, see the text. (1) di-methyl phthalate (DMP), (2) di-ethyl phthalate (DEP), (3) di-isobutyl phthalate (DiBP), (4) di-*n*-butyl phthalate (DBP), (5) bisphenol-A (BPA), (6) bis-(2-ethylhexyl) phthalate (DEHP), (7) di-*n*-octyl phthalate (DNOP), I.S. Internal Standard (phenanthrene).

The analytical protocol was validated in terms of linearity range, correlation coefficients, reproducibility, intra- and inter-day errors and recoveries, and by performing the entire procedure on honey samples (i.e., 2.5 g of the honey sample, addition of 7.5 μL of I.S. 50 pg μL^−1^, 75 μL volume of benzene, pH 4 of the solution, NaCl 10 g L^−1^, vortex 5 min, ultrasounds 6 min, and centrifugation 30 min at 4000 rpm). The proposed method does not have a clean-up, as recently shown in a previous paper where acaricides were determined in honey samples: recoveries can be considered quantitative [35]. Figure 1 shows typical chromatograms of (a) the standard mixture solution (each PAE and BPA at 50 ng g^−1^), (b) the honey sample, and (c) the sample honey spiked with the standard mixture solution. All samples were subjected to the whole procedure: as it can be seen, no peak overlapping was present, the interferences did not affect the qualitative and quantitative analysis, and the peaks were well shaped and clear. Table 5 reports all the analytical data.

Looking overall at the analytical data, the method is robust. In fact, the regression equation and the correlation coefficients (seven-point calibration curve) were good for all compounds in the concentration range analyzed. LODs and LOQs were directly determined in the matrices investigated according to the International Conference on Harmonization: Validation of Analytical Procedure [36] (i.e., an analyte that produces a chromatographic peak equal to three times (LOD) or seven times (LOQ) the standard deviation of the baseline noise). These are sufficient for determining such compounds in honey, according to the regulations 10/2010 and 213/2018 [37,38], which define the specific migration limit (SML) of each PAE/BPA from the plastic into the food (i.e., 60 mg kg^−1^ for DMP, DEP, DiBP, and DNOP, 1.5 mg kg^−1^ for DEHP, 0.3 mg kg^−1^ for DBP and 0.05 mg kg^−1^ for BPA). The intra- and inter-day precision (as relative standard deviation, RSD) were below 7.2 and 9.3, respectively, meaning that the analytical procedure is accurate. Finally, to complete the protocol validation, the recoveries were studied at two different PAE/BPA concentrations (i.e., low (50 ng g^−1^) and high (500 ng g^−1^) fortification). The recoveries ranged between 71–97 % with a RSD below 9 and 76–100 % with a RSD below 6 for low and high fortification, respectively. This was further confirmation about the robustness of the developed analytical protocol for determining PAEs and BPA in the honey matrix.

To highlight the strengths of this paper, a comparison with previous studies [39,40,41,42,43,44,45,46,47,48] performed on such compounds in similar honey matrices is reported in Table 6 in terms of recovery, LOD, LOQ, and RSD whereas three other papers [47,48,49] determined the PAE/BPA concentrations in the honey matrix without describing the analytical parameters. First, the papers showing only analysis are very few in number and only one [six deal with the simultaneous determination of PAEs and BPA. This confirms the importance and difficulties of this issue. As can be seen, the protocol developed in this study allows for the simultaneous investigation of these dangerous compounds at ng g^−1^ levels. The only other paper allowing this simultaneous determination showed higher recoveries (up to 120%) and LODs/LOQs (up to 303 ng g^−1^ and 1013 ng g^−1^).

The procedure was applied to six different honey samples (i.e., four single-use commercial ones and two home-made ones). All samples were stored at 4 °C in darkness. As can be seen in Table 7, the values of the analytes found in the real samples had very low concentrations, in the order of ng g^−1^ and were within the legal limits (as SMLs) established by the European Union. It is quite interesting because in Sample F, the honey was withdrawn from a plastic honeycomb. The use of these kinds of beehives are widely expanding for different reasons, mainly to save the crop or to avoid the wax melting during the hottest periods of the year. Between the two home-made honeys, which were collected in two different areas but not so far from them, the PAE profile seems to be the same, except with a slight enhancement in the BPA level.

Finally, the four commercial samples were subjected to thermal stress: the hypothesis is that high temperatures and irradiation as well as exposure to the Sun’s ultraviolet rays, can break down plastics and cause problems (see Table 8). The main consideration with regard to the DMP is that it seems to have disappeared from all samples (i.e., levels < LOD), whereas the other PAE concentrations remained almost constant (only DEHP increased in Samples B, D, and E). Particular attention should be pointed out for BPA: its level was worryingly increased in Sample D (more than six times) and in Sample B (more than 3-times). Furthermore, after the thermal stress, Sample B showed a BPA content of 0.54 μg g^−1^, which was higher than the current legal limit [38]. This can be explained because the package was produced and distributed before the entry into force of the aforementioned EC Regulation.

Analyses performed after 48 h did not show any significant analytical increases when compared to concentrations obtained after 24 h. This suggests that the significant release of these molecules occurs in the first moments of thermal shock.

## 5. Conclusions

In this paper, an experimental analytical method was developed for the determination of PAEs and BPA from plastic containers inside which honey is contained. The study showed a good robustness of the method, simplicity of execution by the operator, and relatively short analysis times. In addition, the good reproducibility, sensitivity, the use of small quantities of solvents, the short extraction time, and the use of instrument now present in most chemical analysis laboratories, make it easily applicable everywhere. In addition, the analysis of real samples has shown the presence of small quantities of phthalates all below the legal limits currently known.

Finally, the samples subjected to thermal stress for 24 and 48 h showed a slight release only in the first hours. Subsequently, the concentration of the analyzed analytes remained constant. The plastics must be kept out of sunlight and should also avoid high temperatures: our recommendation is to avoid plastic materials not labeled as “Food Safe” or “BPA” free.

## Figures and Tables

**Table 1:** List of compounds investigated in this study. Abbreviations–CAS numbers, formulas, molecular weights (MW), selected ion monitoring (SIM) peaks, solubilities and log K_ow_, median lethal doses (LD_50_), acceptable daily intakes (ADIs).

Compound (Abbrev.—CAS#)	Formula	MW SIM	Solubility ^a^ log K_ow_^b^	LD_50_ (g kg^−1^ mouse)	ADI (ng kg^−1^ bw)
Di-methyl phthalate (DMP—131-11-3)	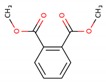	194.18 163, 194	4000 mg L^−1^ 1.60	8–10	79.1
Di-ethyl phthalate (DEP—84-66-2)	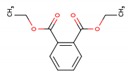	222.24 149, 177	1080 mg L^−1^ 2.42	8–10	1.4–28.2
Di-isobutyl phthalate (DIBP—84-69-5)	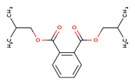	278.34 149, 223	1 mg L^−1^ 4.11	8–10	10^5^
Di-*n*-butyl phthalate (DBP—84-74-2)	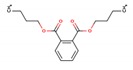	278.35 149, 205	11.2 mg L^−1^ 4.50	8–10	191.8
Bisphenol-A (BP-A—80-05-7)	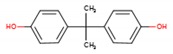	228.29 213, 228	300 mg L^−1^ 3.44	5	69 × 10^6^
Bis-(2-ethylhexyl) phthalate (DEHP—118-81-7)	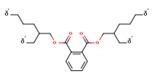	390.56 149, 167	0.09 mg L^−1^ 8.39	14	1458
Di-*n*-octyl phthalate (DNOP—117-84-0)	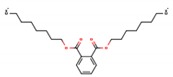	390.56 149, 279	0.022 mg L^−1^ 8.06	13	37 × 10^6^

^a^ Solubility in water at 25 °C [23]; ^b^ the log of the octanol–water partition coefficients.

**Table 2:** Effect of the five different extractant solvents on the PAE/BPA recoveries (% ± standard deviation). Recoveries were obtained by spiking 2.5 g of the honey sample with 200 ng g^−1^ of each PAE/BPA and adding 7.5 μL of I.S. (C_14_, 50 pg μL^−1^), NaCl 10 g L^−1^, and 200 μL volume of the extractant solvent.

	*n*-Heptane	*iso*-Octane	Benzene	Toluene	Tol. + Ac ^1^
DMP	18.6 ± 8.4	17.5 ± 8.1	92.2 ± 6.2	91.0 ± 5.7	112.9 ± 4.8
DEP	89.3 ± 6.4	64.4 ± 10.5	97.6 ± 5.7	96.7 ± 1.9	87.9 ± 7.2
DiBP	109.6 ± 3.7	109.8 ± 2.2	106.9 ± 2.6	110.8 ± 2.9	118.9 ± 6.8
DBP	116.7 ± 4.6	108.6 ± 2.6	101.3 ± 3.4	109.3 ± 0.9	95.6 ± 6.4
BP-A	17.4 ± 3.0	23.5 ± 3.1	74.2 ± 2.7	50.2 ± 5.3	35.6 ± 4.1
DEHP	117.3 ± 7.9	112.6 ± 1.8	105.6 ± 9.9	108.8 ± 4.6	64.0 ± 11.6
DNOP	123.1 ± 6.3	113.4 ± 8.6	103.6 ± 8.8	105.0 ± 7.4	66.0 ± 9.6

^1^ toluene + acetone (1 + 1 v/v).

**Table 3:** Effect of different benzene volumes on the PAE/BPA recoveries (% ± standard deviation). Recoveries were obtained by spiking 2.5 g of the honey sample with 200 ng g^−1^ of each PAE/BPA and adding 7.5 μL of I.S. (C_14_, 50 pg μL^−1^), NaCl 10 g L^−1^, and different volumes of benzene as the extractant solvent.

	25 μL	50 μL	75 μL	100 μL	200 μL
DMP	64.8 ± 3.4	88.8 ± 5.4	101.3 ± 4.5	100.8 ± 9.6	92.2 ± 6.2
DEP	74.5 ± 5.3	87.8 ± 4.8	103.5 ± 4.4	108.1 ± 8.2	97.6 ± 5.7
DiBP	82.6 ± 4.9	100.1 ± 5.1	104.8 ± 7.0	106.3 ± 9.0	106.9 ± 2.6
DBP	98.8 ± 2.5	109.7 ± 2.3	107.0 ± 6.5	91.0 ± 4.1	101.3 ± 3.4
BP-A	24.2 ± 5.1	40.3 ± 6.2	80.6 ± 6.5	70.8 ± 9.1	74.2 ± 2.7
DEHP	87.7 ± 6.8	104.3 ± 5.7	99.5 ± 5.0	96.1 ± 11.8	105.6 ± 9.9
DNOP	84.7 ± 5.1	98.7 ± 7.2	98.5 ± 6.1	91.5 ± 6.4	103.6 ± 8.8

**Table 4:** Effect of pH on the PAE/BPA recoveries (%). Recoveries were obtained by spiking 2.5 g of the honey sample with 200 ng g^−1^ of each PAE/BPA and adding 7.5 μL of I.S. (C_14_, 50 pg μL^−1^), NaCl 10 g L^−1^, and 75 μL volume of benzene.

	pH 4	pH 5	pH 6	pH 7	pH 8
DMP	98.0	92.0	81.7	84.3	74.6
DEP	102.3	97.8	88.3	82.1	76.8
DiBP	104.9	100.6	95.4	88.3	85.4
DBP	102.2	98.7	98.7	87.4	82.7
BP-A	73.1	54.1	44.7	30.5	26.0
DEHP	97.6	93.9	91.4	92.1	91.5
DNOP	103.1	94.3	92.2	89.7	89.4

**Table 5:** Regression equation, correlation coefficient (R^2^) in the range from 50 to 5000 ng g^−1^, limit of detection (LOD) and limit of quantification (LOQ) (ng g^−1^), intra- and inter-day precision (as relative standard deviation, RSD), and recoveries at two different concentrations (low, 50 ng g^−1^, and high, 500 ng g^−1^, fortification) of each PAE and BPA investigated in this study. The selected ion monitoring (SIM) *m/z* of the typical fragment ion for each compound is reported in Table 1.

	Regr. eq. ^a^	R^2^	LOD	LOQ	Intra-Day	Inter-Day	Recovery ^b^	
	(m, q)						50 ng g^−1^	500 ng g^−1^
DMP	0.255, 0.008	0.9973	10	22	3.1	6.4	97.1 (8)	99.8 (5)
DEP	0.263, 0.015	0.9970	7	17	4.7	7.7	96.2 (7)	98.4 (4)
DiBP	0.539, 0.079	0.9963	3	7	5.9	8.2	94.2 (9)	98.6 (6)
DBP	0.625, 0.087	0.9972	4	8	2.4	4.5	93.5 (9)	97.9 (4)
BP-A	0.321, 0.009	0.9996	11	22	7.2	9.3	71.5 (8)	76.2 (5)
DEHP	0.448, 0.062	0.9951	10	19	3.6	7.3	94.6 (8)	99.2 (5)
DNOP	0.720, 0.019	0.9984	13	22	4.5	6.9	97.0 (5)	100.4 (3)

^a^ Regression equation: y = mx + q. ^b^ The RSD is in brackets.

**Table 6:** Comparison among the different analytical data reported in the literature with those found in this study (N/A: data not available in the paper). The matrices are nectar honeys, commercial honeys, and royal jelly.

Methodology	Compounds	Recovery	LOD/LOQ	RSD	Ref.
		(%)	(ng g^−1^)	(%)	
MISPE-LC-DAD-UV ^a^	BPA	N/A	2000	7–12.5	[39]
RAM-LC-MS/MS ^b^	BPA	100–112	9.6/16.6	2.5–11	[40]
RAM-SPE-CE-ESI-MS	BPA	96–103	7.5/N/A	<22	[41]
LLE-GC-MS	DBP, DMP, DEP, DEHP, DiBP, DNOP	80.1–110.9	0.3–6.0/10–17.5	<11.8	[42]
DLLME-GC-FID	BPA	91–101	16–47/14–48	<7.5	[43]
SPE-GC-MS	BPA, DBP, DMP, DEP, DEHP, DiBP	81.2–119.8	5–303/12–1013	1.5–4.1	[44]
SPE-GC-MS	BPA	103	0.128/0.428	4.9–10.2	[45]
ST-DLLME-HPLC-PAD ^c^	DBP	N/A	150/500	N/A	[46]
This study	DMP, DEP, DiBP, DBP, BPA, DEHP, DNOP	71.5–100.4	2–13/7–22	3–9	

^a^ MISPE-LC-DAD-UV: molecularly imprinted solid phase extraction-liquid chromatography-diode array detection. ^b^ RAM-LC-MS/MS: restricted-access material-LC/MS/MS. ^c^ ST-DLLME-HPLC-PAD: solvent terminated DLLME-high performance liquid chromatography-photodiode array detector.

**Table 7:** PAE and BPA levels (µg g^−1^) found in the six home-made and commercial honey samples analyzed by the proposed protocol.

Sample ^1^	A	B	C	D	E	F
DMP	<LOD	0.03	0.04	<LOD	<LOD	<LOD
DEP	<LOD	0.02	5.05	<LOD	<LOD	<LOD
DiBP	0.02	<LOD	0.26	0.05	<LOD	0.01
DBP	0.08	<LOD	0.28	0.05	<LOD	0.05
BP-A	0.06	0.15	0.02	0.04	0.19	0.27
DEHP	0.20	0.10	0.84	0.30	<LOD	0.19
DNOP	0.20	0.02	0.72	0.18	<LOD	0.21

^1^ A: home-made honey; B–E: commercial; F: home-made from plastic honeycomb.

**Table 8:** PAE and BPA levels (µg g^−1^) found in the commercial honey samples subjected to thermal stress (24 h at 40 °C).

Sample ^1^	B	C	D	E
DMP	<LOD	<LOD	<LOD	<LOD
DEP	<LOD	5.05	0.71	<LOD
DiBP	<LOD	0.26	<LOD	0.02
DBP	<LOD	0.28	<LOD	0.03
BP-A	0.54	0.02	0.26	0.19
DEHP	0.18	0.84	0.36	0.24
DNOP	0.04	0.72	<LOD	0.20

^1^ B–E: commercial honey.
